# 5-Carb­oxy-2,4-dihy­droxy­anilinium chloride dihydrate

**DOI:** 10.1107/S1600536811000559

**Published:** 2011-01-08

**Authors:** Syeda Sohaila Naz, Nazar Ul Islam, M. Nawaz Tahir

**Affiliations:** a Institute of Chemical Sciences, University of Peshawar, Peshawar, Pakistan; b Department of Physics, University of Sargodha, Sargodha, Pakistan

## Abstract

In the title compound, C_7_H_8_NO_4_^+^·Cl^−^·2H_2_O, the organic mol­ecule is almost planar with an r.m.s. deviation of 0.0164 Å for all non-H atoms. An *S*(6) ring motif is formed due to an intra­molecular O—H⋯O hydrogen bond. In the crystal, the mol­ecules are linked into a three-dimensional network by N—H⋯Cl, N—H⋯O, O—H⋯Cl and O—H⋯O hydrogen bonds.

## Related literature

For a related structure, see: Naz *et al.* (2010 ▶). For graph-set notation, see: Bernstein *et al.* (1995 ▶).
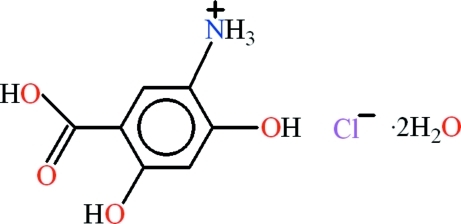


## Experimental

### 

#### Crystal data

C_7_H_8_NO_4_^+^·Cl^−^·2H_2_O
*M* *_r_* = 241.63
Triclinic, 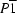

*a* = 6.0285 (8) Å
*b* = 7.9597 (8) Å
*c* = 10.9570 (13) Å
α = 100.135 (5)°
β = 97.162 (4)°
γ = 92.921 (5)°
*V* = 512.10 (11) Å^3^
*Z* = 2
Mo *K*α radiation
μ = 0.38 mm^−1^
*T* = 296 K
0.28 × 0.15 × 0.10 mm

#### Data collection

Bruker Kappa APEXII CCD diffractometer
Absorption correction: multi-scan (*SADABS*; Bruker, 2005 ▶) *T*_min_ = 0.935, *T*_max_ = 0.965
8850 measured reflections
2548 independent reflections
1853 reflections with *I* > 2σ(*I*)
*R*_int_ = 0.039

#### Refinement

*R*[*F* ^2^ > 2σ(*F* ^2^)] = 0.044
*wR*(*F* ^2^) = 0.115
*S* = 1.03
2548 reflections
139 parameters
H-atom parameters constrained
Δρ_max_ = 0.40 e Å^−3^
Δρ_min_ = −0.27 e Å^−3^

### 

Data collection: *APEX2* (Bruker, 2009 ▶); cell refinement: *SAINT* (Bruker, 2009 ▶); data reduction: *SAINT*; program(s) used to solve structure: *SHELXS97* (Sheldrick, 2008 ▶); program(s) used to refine structure: *SHELXL97* (Sheldrick, 2008 ▶); molecular graphics: *ORTEP-3 for Windows* (Farrugia, 1997 ▶) and *PLATON* (Spek, 2009 ▶); software used to prepare material for publication: *WinGX* (Farrugia, 1999 ▶) and *PLATON*.

## Supplementary Material

Crystal structure: contains datablocks global, I. DOI: 10.1107/S1600536811000559/dn2650sup1.cif

Structure factors: contains datablocks I. DOI: 10.1107/S1600536811000559/dn2650Isup2.hkl

Additional supplementary materials:  crystallographic information; 3D view; checkCIF report

## Figures and Tables

**Table 1:** Hydrogen-bond geometry (Å, °)

*D*—H⋯*A*	*D*—H	H⋯*A*	*D*⋯*A*	*D*—H⋯*A*
O1—H1⋯O6	0.82	1.88	2.6945 (19)	171
O3—H3⋯O2	0.82	1.96	2.672 (2)	145
O4—H4*A*⋯Cl1^i^	0.82	2.21	3.0097 (15)	164
N1—H1*A*⋯O6^ii^	0.89	2.02	2.903 (2)	169
N1—H1*B*⋯O5^iii^	0.89	1.96	2.853 (2)	178
N1—H1*C*⋯Cl1^iv^	0.89	2.35	3.1950 (18)	157
O5—H5*A*⋯O2^v^	0.86	2.16	2.935 (2)	149
O5—H5*B*⋯Cl1	0.85	2.41	3.1494 (15)	146
O6—H6*A*⋯Cl1	0.88	2.27	3.1386 (16)	174
O6—H6*B*⋯O5^vi^	0.86	1.99	2.850 (2)	173

Symmetry codes: (i) 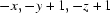; (ii) 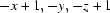; (iii) 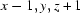; (iv) 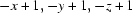; (v) 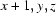; (vi) 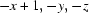.

**Fig. 1.**
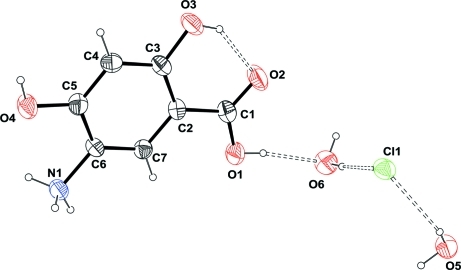
View of the title compound with the atom numbering scheme. The displacement ellipsoids are drawn at the 50% probability level. H-atoms are shown as small spheres of arbitrary radii. The dotted line represents the intramolecular H-bonding.

**Fig. 2.**
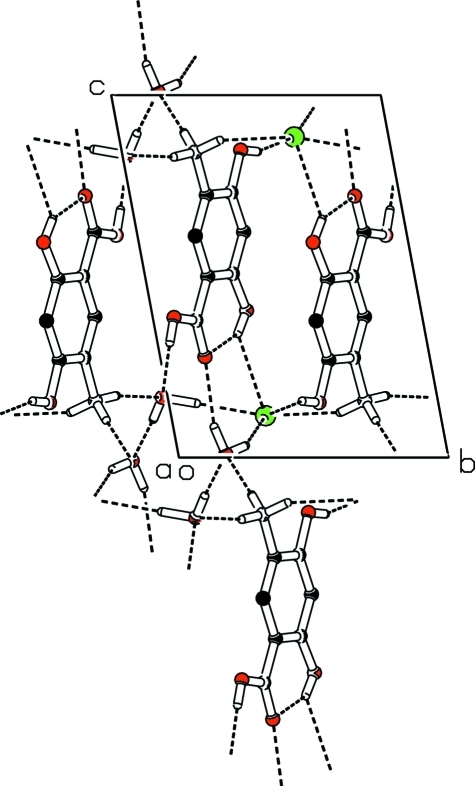
The partial packing (PLATON; Spek, 2009) which shows that molecules form various ring motifs and molecules are interlinked through H-bondings to form three dimensional polymeric network.

## supplementary crystallographic information

### Comment

Recently we have reported the crystal structure of 5-carboxy-2,4-dihydroxyanilinium chloride (Naz *et al.*, 2010). The title compound (I, Fig. 1) has been prepared in a slightly different way.

In (I), the organic group (C1—C7/O1—O4/N1) is planar with r. m. s. deviation of 0.0164 Å. In the organic part, there exist a strong intramolecular H-bond of O—H···O type (Table 1, Fig. 1) completing an S(6) ring motif (Bernstein *et al.*, 1995). In the title compound, the Cl^-^ anion is penta coordinated due to H-bondings of N—H···Cl and O—H···Cl types (Table 1, Fig. 2). The NH_3_^+^ ion makes H-bonding with the both water molecules and the Cl^-^ ion. Due to these strong H-bondings the molecules are stabilized in the form of three-dimensional polymeric network (Table 1, Fig. 2). There does not exist any π···π or C—H···π interaction.

### Experimental

Concentrated nitric acid (2 mL, 67%) was added drop by drop to β-resorcylic acid (1 g, 97%, 6.3 mmol) in a round bottom flask. The mixture was protected from moisture by CaCl_2_ (anhydrous) tube and was allowed to stand for 12 h at room temperature. Then reaction mixture was diluted with water. The crude material was filtered and recrystallized from water to affoard the 5-nitro-β-resorcylic acid.

Then a mixture of 5-nitro-β-resorcylic acid (1.5 g, 7.5 mmol), tin (3 g, 25 mmol) and absolute ethanol (5 ml) were taken in a 100 ml round bottom flask and passed HCl gas under reflux with stirring for 1 h. The completion of reaction was monitored by TLC. The reaction mixture was filtered to remove any unreacted tin. Filtrate was kept for seven days to afford light brown needles of (I).

### Refinement

All H atoms attached to C atoms, N atom and hydroxyl O atoms were fixed geometrically and treated as riding with C—H = 0.93 Å (C), N—H = 0.89 Å and

O-H = 0.82Å with U_iso_(H) = 1.2U_eq_(C) and U_iso_(H) = 1.5U_eq_(N,O). H atoms of water molecules were located in difference Fourier maps and included in the subsequent refinement using restraints (O-H= 0.85 (1)Å and H···H= 1.40 (2)Å) with U_iso_(H) = 1.5U_eq_(O). In the last cycles of refinement, they were treated as riding on their parent O atoms.

### Crystal data

**Table:**

C_7_H_8_NO_4_^+^·Cl^−^·2H_2_O	*Z* = 2
*M**_r_* = 241.63	*F*(000) = 252
Triclinic, *P*1	*D*_x_ = 1.567 Mg m^−^^3^
Hall symbol: -P 1	Mo *K*α radiation, λ = 0.71073 Å
*a* = 6.0285 (8) Å	Cell parameters from 1853 reflections
*b* = 7.9597 (8) Å	θ = 1.9–28.4°
*c* = 10.9570 (13) Å	µ = 0.38 mm^−^^1^
α = 100.135 (5)°	*T* = 296 K
β = 97.162 (4)°	Needle, brown
γ = 92.921 (5)°	0.28 × 0.15 × 0.10 mm
*V* = 512.10 (11) Å^3^	

### Data collection

**Table:**

Bruker Kappa APEXII CCD diffractometer	2548 independent reflections
Radiation source: fine-focus sealed tube	1853 reflections with *I* > 2σ(*I*)
graphite	*R*_int_ = 0.039
Detector resolution: 7.50 pixels mm^-1^	θ_max_ = 28.4°, θ_min_ = 1.9°
ω scans	*h* = −8→8
Absorption correction: multi-scan (*SADABS*; Bruker, 2005)	*k* = −10→8
*T*_min_ = 0.935, *T*_max_ = 0.965	*l* = −14→14
8850 measured reflections	

### Refinement

**Table:**

Refinement on *F*^2^	Primary atom site location: structure-invariant direct methods
Least-squares matrix: full	Secondary atom site location: difference Fourier map
*R*[*F*^2^ > 2σ(*F*^2^)] = 0.044	Hydrogen site location: inferred from neighbouring sites
*wR*(*F*^2^) = 0.115	H-atom parameters constrained
*S* = 1.03	*w* = 1/[σ^2^(*F*_o_^2^) + (0.0571*P*)^2^ + 0.0225*P*] where *P* = (*F*_o_^2^ + 2*F*_c_^2^)/3
2548 reflections	(Δ/σ)_max_ < 0.001
139 parameters	Δρ_max_ = 0.40 e Å^−^^3^
0 restraints	Δρ_min_ = −0.27 e Å^−^^3^

### Special details

**Table:**

Geometry. All esds (except the esd in the dihedral angle between two l.s. planes) are estimated using the full covariance matrix. The cell esds are taken into account individually in the estimation of esds in distances, angles and torsion angles; correlations between esds in cell parameters are only used when they are defined by crystal symmetry. An approximate (isotropic) treatment of cell esds is used for estimating esds involving l.s. planes.
Refinement. Refinement of *F*^2^ against ALL reflections. The weighted *R*-factor *wR* and goodness of fit *S* are based on *F*^2^, conventional *R*-factors *R* are based on *F*, with *F* set to zero for negative *F*^2^. The threshold expression of *F*^2^ > σ(*F*^2^) is used only for calculating *R*-factors(gt) *etc*. and is not relevant to the choice of reflections for refinement. *R*-factors based on *F*^2^ are statistically about twice as large as those based on *F*, and *R*- factors based on ALL data will be even larger.

### Fractional atomic coordinates and isotropic or equivalent isotropic displacement parameters (Å^2^)

**Table:**

	*x*	*y*	*z*	*U*_iso_*/*U*_eq_	
O1	0.2807 (3)	0.0789 (2)	0.38672 (13)	0.0395 (4)	
H1	0.3127	0.0453	0.3164	0.059*	
O2	−0.0155 (3)	0.1663 (2)	0.27792 (13)	0.0454 (4)	
O3	−0.2957 (3)	0.35281 (19)	0.40596 (13)	0.0401 (4)	
H3	−0.2501	0.3044	0.3424	0.060*	
O4	−0.1327 (3)	0.4424 (2)	0.84841 (13)	0.0415 (4)	
H4A	−0.2380	0.5033	0.8442	0.062*	
N1	0.2345 (3)	0.2711 (2)	0.84317 (14)	0.0306 (4)	
H1A	0.3302	0.1906	0.8294	0.046*	
H1B	0.1449	0.2439	0.8965	0.046*	
H1C	0.3104	0.3712	0.8755	0.046*	
C1	0.0937 (4)	0.1574 (2)	0.37826 (18)	0.0303 (5)	
C2	0.0302 (3)	0.2322 (2)	0.50004 (17)	0.0268 (4)	
C3	−0.1622 (4)	0.3252 (2)	0.50731 (18)	0.0284 (4)	
C4	−0.2215 (3)	0.3963 (2)	0.62285 (18)	0.0303 (5)	
H4	−0.3489	0.4570	0.6270	0.036*	
C5	−0.0912 (3)	0.3766 (2)	0.73114 (18)	0.0286 (5)	
C6	0.0990 (3)	0.2839 (2)	0.72445 (17)	0.0248 (4)	
C7	0.1595 (3)	0.2145 (2)	0.61147 (17)	0.0259 (4)	
H7	0.2879	0.1547	0.6086	0.031*	
O5	0.9425 (3)	0.17641 (17)	0.00957 (13)	0.0372 (4)	
H5A	0.9959	0.1488	0.0794	0.056*	
H5B	0.8641	0.2602	0.0303	0.056*	
O6	0.4319 (3)	−0.02049 (19)	0.16590 (14)	0.0427 (4)	
H6A	0.4697	0.0818	0.1525	0.064*	
H6B	0.3236	−0.0641	0.1078	0.064*	
Cl1	0.53107 (9)	0.35035 (6)	0.11554 (5)	0.03442 (17)	

### Atomic displacement parameters (Å^2^)

**Table:**

	*U*^11^	*U*^22^	*U*^33^	*U*^12^	*U*^13^	*U*^23^
O1	0.0407 (10)	0.0515 (9)	0.0269 (8)	0.0127 (7)	0.0097 (7)	0.0025 (7)
O2	0.0577 (11)	0.0566 (10)	0.0220 (8)	0.0189 (8)	0.0017 (7)	0.0056 (7)
O3	0.0422 (10)	0.0517 (9)	0.0257 (8)	0.0121 (7)	−0.0042 (7)	0.0093 (7)
O4	0.0478 (10)	0.0534 (10)	0.0251 (8)	0.0274 (8)	0.0074 (7)	0.0035 (7)
N1	0.0326 (10)	0.0357 (9)	0.0231 (9)	0.0089 (7)	0.0018 (7)	0.0043 (7)
C1	0.0377 (13)	0.0276 (10)	0.0255 (11)	0.0000 (9)	0.0033 (9)	0.0058 (8)
C2	0.0325 (12)	0.0258 (9)	0.0229 (10)	0.0025 (8)	0.0059 (9)	0.0050 (8)
C3	0.0319 (12)	0.0284 (10)	0.0246 (11)	0.0018 (8)	−0.0012 (9)	0.0081 (8)
C4	0.0282 (11)	0.0322 (10)	0.0323 (12)	0.0097 (8)	0.0035 (9)	0.0086 (9)
C5	0.0327 (12)	0.0271 (10)	0.0271 (11)	0.0052 (8)	0.0059 (9)	0.0062 (8)
C6	0.0289 (11)	0.0242 (9)	0.0205 (10)	0.0036 (8)	0.0000 (8)	0.0038 (8)
C7	0.0272 (11)	0.0251 (9)	0.0261 (11)	0.0037 (8)	0.0049 (8)	0.0047 (8)
O5	0.0415 (9)	0.0386 (8)	0.0343 (8)	0.0153 (7)	0.0085 (7)	0.0085 (7)
O6	0.0468 (10)	0.0400 (8)	0.0408 (9)	0.0073 (7)	0.0065 (8)	0.0045 (7)
Cl1	0.0335 (3)	0.0385 (3)	0.0323 (3)	0.0111 (2)	0.0022 (2)	0.0086 (2)

### Geometric parameters (Å, °)

**Table:**

O1—C1	1.317 (2)	C2—C7	1.397 (3)
O1—H1	0.8200	C2—C3	1.410 (3)
O2—C1	1.225 (2)	C3—C4	1.390 (3)
O3—C3	1.346 (2)	C4—C5	1.376 (3)
O3—H3	0.8199	C4—H4	0.9300
O4—C5	1.358 (2)	C5—C6	1.397 (3)
O4—H4A	0.8200	C6—C7	1.365 (3)
N1—C6	1.470 (2)	C7—H7	0.9300
N1—H1A	0.8900	O5—H5A	0.8616
N1—H1B	0.8900	O5—H5B	0.8517
N1—H1C	0.8900	O6—H6A	0.8764
C1—C2	1.467 (3)	O6—H6B	0.8647
			
C1—O1—H1	109.5	O3—C3—C2	123.24 (18)
C3—O3—H3	109.5	C4—C3—C2	120.50 (18)
C5—O4—H4A	109.5	C5—C4—C3	119.87 (18)
C6—N1—H1A	109.5	C5—C4—H4	120.1
C6—N1—H1B	109.5	C3—C4—H4	120.1
H1A—N1—H1B	109.5	O4—C5—C4	124.58 (17)
C6—N1—H1C	109.5	O4—C5—C6	115.57 (17)
H1A—N1—H1C	109.5	C4—C5—C6	119.85 (18)
H1B—N1—H1C	109.5	C7—C6—C5	120.75 (18)
O2—C1—O1	122.79 (18)	C7—C6—N1	121.88 (16)
O2—C1—C2	123.65 (19)	C5—C6—N1	117.31 (16)
O1—C1—C2	113.56 (17)	C6—C7—C2	120.62 (17)
C7—C2—C3	118.40 (17)	C6—C7—H7	119.7
C7—C2—C1	120.94 (18)	C2—C7—H7	119.7
C3—C2—C1	120.67 (18)	H5A—O5—H5B	104.7
O3—C3—C4	116.25 (17)	H6A—O6—H6B	106.8

### Hydrogen-bond geometry (Å, °)

**Table:**

*D*—H···*A*	*D*—H	H···*A*	*D*···*A*	*D*—H···*A*
O1—H1···O6	0.82	1.88	2.6945 (19)	171
O3—H3···O2	0.82	1.96	2.672 (2)	145
O4—H4A···Cl1^i^	0.82	2.21	3.0097 (15)	164
N1—H1A···O6^ii^	0.89	2.02	2.903 (2)	169
N1—H1B···O5^iii^	0.89	1.96	2.853 (2)	178
N1—H1C···Cl1^iv^	0.89	2.35	3.1950 (18)	157
O5—H5A···O2^v^	0.86	2.16	2.935 (2)	149
O5—H5B···Cl1	0.85	2.41	3.1494 (15)	146
O6—H6A···Cl1	0.88	2.27	3.1386 (16)	174
O6—H6B···O5^vi^	0.86	1.99	2.850 (2)	173

Symmetry codes: (i) −*x*, −*y*+1, −*z*+1; (ii) −*x*+1, −*y*, −*z*+1; (iii) *x*−1, *y*, *z*+1; (iv) −*x*+1, −*y*+1, −*z*+1; (v) *x*+1, *y*, *z*; (vi) −*x*+1, −*y*, −*z*.
